# Immunomodulatory Effects of Danshen (*Salvia miltiorrhiza*) in BALB/c Mice

**DOI:** 10.5402/2012/954032

**Published:** 2012-10-16

**Authors:** Donghong Gao, Alvaro Mendoza, Shijun Lu, David A. Lawrence

**Affiliations:** Biggs Laboratory, Wadsworth Center, New York State Department of Health, Albany, NY 12208, USA

## Abstract

Danshen, the root and rhizome of *Salvia miltiorrhiza* Bge, a Traditional Chinese Medicine, especially for cardiovascular and cerebrovascular diseases, has unique immunomodulatory effects. Danshen is capable of anti-inflammation and antiallergy, which are immunosuppressive activities, whereas it is also able to promote immunity against cancer, viruses, and bacteria. Most previous reports were performed with use of a purified compound or compounds of Danshen. Since there are more than twenty active compounds in Danshen, it is very difficult to predict that one compound will act the same way when it is combined with other compounds. In order to overcome this limitation, we used the crude form of Danshen to study its immunomodulatory effects in a mouse model. The mice were fed daily diet supplements of Danshen for three months and then tested for their immunity, including leukocyte subsets in peripheral blood, humoral and cell-mediated immune responses, and host defenses against a *Listeria monocytogenes (LM)* infection. Different doses of Danshen caused different immunomodulatory effects. Danshen at 0.5% decreased serum IgE production in BALB/c mice; 1% Danshen promoted cell-mediated immunity; Danshen at 0.5 and 1% inhibited the production of oxygen free radicals in liver and spleen and NO production in liver; 2% Danshen enhanced the host resistance against *LM* with increased numbers of peripheral monocytes and natural killer (NK) cells and decreased production of IL-1**β** and NO.

## 1. Introduction

Complementary and alternative medicine (CAM) is defined as any healing practice other than conventional medicine [1]. It includes naturopathy, chiropractic, herbalism, Traditional Chinese Medicine (TCM), yoga, acupuncture, diet-based therapies, and many other practices. The techniques in alternative medicine have been around for thousands of years. They have been widely used and taught in eastern countries. Now, people in western countries are more willing to try alternative medicine.

Danshen belongs to the CAM category. It is the root and rhizome of *Salvia miltiorrhiza* Bge. It has been a TCM for at least two thousand years. The major functions of this herb in TCM are *huo xue hua yu* (activating blood circulation to disperse stasis), *jie du xiao zhong* (removing toxic substances and promoting subsidence of swelling), and *qing xin an shen* (nourishing the heart to calm the mind, tranquilizing the mind by nourishing the heart) [2]. Traditionally, it has been utilized for treatment of irregular menses, menstrual pain, amenorrhea, precordial pain, abdominal pain, abdominal mass, body and joint pain, carbuncle, furuncle, and skin ulcer, as well as palpitations, fidgetiness, and insomnia [2, 3]. The most common modern uses of this herb are for cardiovascular and cerebrovascular diseases such as angina pectoris, coronary heart disease, myocardial infarction, and stroke [3–5]. An extract of Danshen or *Fufang* Danshen (combined with other herbs) has been used as the standard therapy for cerebral infarction or other ischemic conditions in China [4, 5]. Many new therapies have used Danshen as the control in clinical trials [4, 5]. In addition to its therapeutics for cardiovascular and brain disorders, preparations of Danshen have been shown to have protective effects for liver [3, 6–9], kidney [3, 6, 10, 11], and lung [3] in various experimental models. Although the mechanisms have not been fully delineated, several mechanisms have been proposed to explain the therapeutic or protective capabilities of Danshen. For example, it has been suggested that Danshen has an inhibitory effect on angiotensin-converting enzyme (ACE) to lower blood pressure, and dilate arteries [12, 13]. Therefore, it can decrease the risk of having a stroke and improve ischemic conditions. It has also been suggested that Danshen has anticoagulant capacity, which is beneficial for preventing thrombosis. Unlike other anticoagulant drugs, Danshen's anticoagulation is unique in that it increases the proteolysis of fibrinogen [4] and inhibits platelet aggregation [14], which are suggested to be mediated through stabilizing intracellular calcium and inhibition of arachidonic acid metabolism and thromboxane A_2_ production [15, 16]. Studies of Danshen have also demonstrated that it has antioxidant effects [6–8, 17–22] and anti-inflammatory activities [9, 23–29]. Danshen can induce arterial dilation, clot dissolution and help blood reperfusion; however, oxygen reflowing to the ischemic tissues causes oxidative stress to those tissues since their mitochondrial and cellular enzymes are not fully functional. It is believed that Danshen's antioxidant action lessens the associated problems. Danshen is reported to activate antioxidant defense enzymes such as superoxide dismutase, catalase, glutathione perioxidase, and glutathione S-transferase [8, 9, 17, 21, 22], scavenge oxygen free radicals [18–20, 30, 31], reduce reactive oxygen species (ROS) formation [6, 7, 9, 21], and prevent intracellular glutathione (GSH) depletion [7]. In addition, Liu et al. (2007) reported that Danshen can prevent oxidative stress-induced endothelial cell apoptosis dependent on a PI3K/Akt/Raf/MEK/ERK signaling pathway [32]. Danshen's anti-inflammation activity contributes to its protective effect on organs or cells from excessive inflammation under various pathological conditions. Using *in vitro* and *in vivo* LPS-induced inflammation models, Danshen inhibits release of early (TNF-*α*, IL-1*β*) and late (High Mobility Group Box  1, HMGB 1) proinflammatory cytokines [9, 23, 24, 28, 29]. The inhibitory effect could involve NIK-IKK, ERK1/2, p38, and JNK dependent pathways [25]. *In vitro* data also suggested that Danshen's anti-inflammation effect was related to the inhibition of macrophage chemotaxis, which was mediated through impeding F-actin polymerization, filopodia formation, and negative regulation of PI3K signaling pathway [26, 27].

Since Danshen is chemically complex, in that more than twenty active compounds have been identified [4, 33], and there are no known agonistic or antagonistic interactions amongst those compounds, we believe it is useful to begin testing of Danshen in its crude form, in that it is the rather crude form of Danshen that is closer to its TCM usage. Thus, we have evaluated the immunomodulatory effects of Danshen powder as a supplement to the daily diet of BALB/c mice. After 3-month feeding, we tested the toxicity of Danshen and various immunological responses of the mice, including the host defenses of the mice against a *Listeria monocytogenes* (*LM*) infection. *LM* is a well-defined intracellular pathogen, and innate and adaptive immunity combine to combat this infection [34–36]. We found that different doses of Danshen showed different immunomodulatory effects. Mice ingesting Danshen at different percentages of their daily food intake had differential modulation of various immune responses. Most interesting, the Danshen-induced enhancement of bacterial resistance was correlated with increased numbers of peripheral monocytes and natural killer (NK) cells and decreased the production of IL-1*β* and NO.

## 2. Material and Methods

### 2.1. Mice

2- to 3-month-old male BALB/c mice were obtained from Taconic farms (Taconic, Germantown, NY). Mice were housed in our specified pathogen-free AAALAC-approved facility of the Wadsworth Center and were maintained on mouse chow and acidified water *ad libitum*. All of the studies were IACUC approved.

### 2.2. Mouse Food Preparation

**Figure 1:**
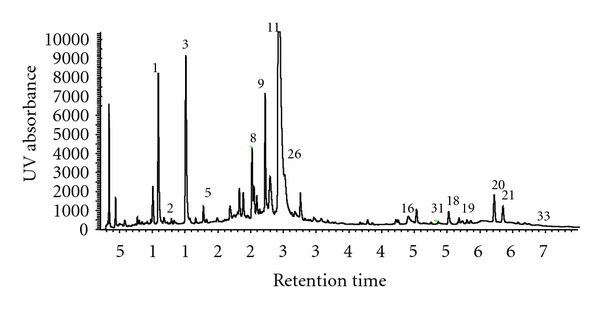
UV chromatogram by HPLC of a methanol : H_2_O (70 : 30, v/v) extract of Danshen peaks which are identified by total ion current (LC-MS) and spectrometric data. Identified constituents are 1, Danshensu; 2, Protocatechuic acid; 3, Procatechu aldehyde; 5, Caffeic acid; 8, Salvianolic acid E; 9, Rosmarinic acid; 10, Lithospermic acid; 11, Salvianolic acid B; 16, Tanshinone IIB; 18, Dihydrotanshinone I; 19, Methyltanshinonate; 20, Cryptotanshinone; 21, Tanshinone I; 26, Isosalvianolic acid B; 31, Neocryptotanshinone; and 33, Methylenetanshinone.

LabDiet 5002 Certified Rodent Diet in meal form (powdered chow) was used as mouse food in this study. The powdered chow (PMI Nutrition International, Brentwood, MO) was mixed with 0, 0.5, 1, and 2% of Danshen powder (w/w), which was purchased from Crane herb Company, Inc (Mashpee, MA). The food was placed in mouse feeding jars. The jars were cleaned and refilled with fresh food twice a week. The commercial Danshen powder was tested for heavy metal content as well as chemical compounds. Danshen had <0.08 *μ*g Hg/g, which was the same amount of Hg as the powdered chow. It also contained 0.4 *μ*g Pb/g, which was 0.2 *μ*g/g in the powdered chow. No other potential toxic metals were detected. Thirty-five different compounds were identified in Danshen powder by HPLC and LC-MS/MS analysis, including Danshensu, Protocatechuic acid, Protocatechu aldehyde, Vanillic acid, Caffeic acid, Ferulic acid, Salvianolic acid D, Salvianolic acid E, Rosmarinic acid, Lithospermic acid, Salvianolic acid B, Salvianolic acid A, Tanshindiol C, Tanshindiol B, Salvianolic acid C, Tanshinone IIB, Przewa tanshinone A, Dihydrotanshinone I, Methyltanshinonate, Cryptotanshinone, Tanshinone I, Diehydromiltirone, Tanshinone IIA, Salvianolic acid F, Salvianolic acid I, Isosalvianolic acid B, Salvianolic acid L, Royleanone-4, Neocryptotanshinone, Methylenetanshinone (Figure 1, an example, chromatogram of the detected Danshen constituents).

### 2.3. Reagents

A stock solution of keyhole limpet hemocyanin (KLH) was purchased from Calbiochem (San Diego, CA). The stock solutions were diluted with sterile saline. Sterile 0.9% NaCl was purchased from Baxter Healthcare Corporation (Deerfield, IL). Sterile 1x PBS was purchased from BioWhittaker (Lonza, Walkerville, MD). Sterile 0.9% Sodium Chloride was purchased from Baxter (Deerfield, IL). Tissue lysis buffer (10x) was made of 500 mM Tris-Cl, 1.5 M NaCl, 20 mM EDTA, 10 mM Na-Orthovanadate, 50 mM NaF, with freshly added 1% NP-40 and cocktail protease inhibitors (chemicals were all purchased from Sigma, St. Louis, MO). Red blood cell lysing buffer was made of 0.017 M Tris and 0.14 M NHCl_4_, pH 7.4. Griess reagent was freshly made of a 1 : 1 mixture of 1% *p*-aminobenzenesulfonamide in 5% H_3_PO_4_ and 0.1% naphthyl ethylenldiamine dihydrochloride in H_2_O. All reagents were confirmed to be endotoxin-free or low endotoxin by Limulus Amebocyte Lysate QCL-1000 (Biowhittaker, Walersville, MD) test before *in vivo* or *in vitro* use.

### 2.4. Immunization

After taking Danshen for 5 weeks, mice were immunized with KLH (100 *μ*g in 100 *μ*L saline) and then boosted with KLH at the 7th week. The blood was collected one week after the second immunization. The serum was stored for IgG1, IgG2a, and IgE analyses.

### 2.5. Serum Preparation

Peripheral blood was obtained by retroorbital phlebotomy into 1.7-mL Eppendorf tubes. After clotting overnight at 4°C, serum was collected after centrifugation.

### 2.6. ELISA for IgG Isotype and IgE

IgG isotype and IgE were measured by a standard ELISA assay as described previously [37].

### 2.7. Cytokine Detection

Cytokine levels of liver or spleen homogenate were detected by using DuoSet ELISA development kits (R&D System, Minneapolis, MN). The manufacturer's protocol was employed. Briefly, the plate was coated with anticytokine monoclonal antibody (mAb). After washing, serial diluted standards and samples were loaded on the plate. After incubation, biotin-labeled anticytokine mAb was applied, followed by avidin-peroxidase and finally the substrate. The plates were read using an ELISA reader (EL310; Bio-Tek, Burlington, VT) at 450 nm [38]. For quantification of high-mobility group box 1 (HMGB 1), the above protocol was utilized with some modifications. Mouse mAb to HMGB1 (ABCAM Limited, Cambridge, MA) was used as the capture antibody (Ab), and rabbit polyclonal antibody (pAb) to HMGB1 (ABCAM Limited, Cambridge, MA) was applied as the detection Ab, followed by goat anti-rabbit IgG whole molecule peroxidase (HRP) conjugated Ab (Sigma, St. Louis, MO), and peptide HMGB 1 (ABCAM Limited, Cambridge, MA) which served as standard. Cytokine production in serum was measured by using Luminex xMAP technology from EMD Millipore (Billerica, MA). The manufacturer's protocol was used. Briefly, serial diluted standards and samples were mixed with various beads which represented different cytokines. After overnight incubation on a Titer plate shaker at 4°C, dark, the plate was washed by using a Vacuum filtration unit. After addition the mixture of biotin labeled anti-cytokine as detection antibodies, the plate was incubated at room temperature for one hour, dark. Then Streptavidin-phycoerythrin was added. After washing, the beads were resuspended in Sheath fluid, and the plate was read on Luminex 100. The data were analyzed by using Upstate Beadview program.

### 2.8. Delayed Type Hypersensitivity (DTH) Assay

DTH assay was performed as described previously [37].

### 2.9. Flow-Cytometric Analysis

Whole blood was prepared and analyzed by multicolor flow cytometry (Becton, Dickinson and Copang., Mountain View, CA). All antibodies were purchased from BD Pharmingen (San Diego, CA). For testing natural killer (NK) or B cells, 1 *μ*g of FITC anti-mouse pan NK, PE anti-mouse CD19, PerCp anti-mouse CD45, and APC anti-mouse CD3 were mixed together and the mixture was added to TruCOUNT tubes (BD Biosciences). Whole blood was mixed and incubated with 1 *μ*g Fc block (anti-CD16/32) first, and then 50 *μ*L of whole blood was added to each TruCOUNT tube containing antibody mixtures. After 30 min incubation at room temperature (RT), 450 *μ*L of 1x BD FACS Lysis was added to each tube. The cells were analyzed on the flow cytometer by gating out the majority of nonviable cells based on low forward angle light scatter. Neutrophil, monocyte, and lymphocyte population were selected based on the side scatter and CD45 staining.

### 2.10. *LM* Infection

The *LM* stock was prepared and stored as previously described [34]. Mice were intravenously (i.v.) injected with a sublethal dose of *LM* (5–8 × 10^3^ colony forming units, CFU). Then the mice were sacrificed three days after inoculation. Liver and spleen were harvested for assessment of bacterial burden and cytokine containing.

### 2.11. Determination of *LM* Burden in Liver and Spleen

Liver and spleen were removed and homogenized in sterile 0.9% NaCl. Then liver and spleen homogenates were aliquoted and mixed with tissue lysis buffer (10x) as 9 : 1 ration and stored at −80°C for further assays. For the enumeration of viable *LM*, serial dilutions of organ homogenates were plated on blood-agar plates. After overnight incubation at 37°C, CFU was counted. When a portion of liver or spleen was collected before the organ was homogenized, the whole organ bacteria burden was calculated back by using the bacteria burden in the remaining fragment divided by the ratio of weight of the remaining fragment to the whole organ.

### 2.12. DMPO Assay

A portion of spleen or liver was weighed and homogenized in tissue lysis buffer. Organ homogenates were immediately treated with 100 mM of spin trapping reagent 5,5-dimetyl-1-pyrroline *N*-oxide (DMPO) (Sigma-Aldrich, St. Louis, MO) and allowed to incubate at room temperature (RT) for 1 h in the dark. The homogenates were centrifuged at 16,000 ×g at 4°C and the supernatants stored at −20°C until analysis. Radical-derived DMPO nitrone adducts were determined using a standard ELISA 96-well plate (Costar, high binding, Corning, Inc., Corning, NY) in which the plate was coated with 4 *μ*g per well of protein solution in 100 *μ*L of coating buffer (100 mM sodium biocarbonate, pH 9.6) and incubated overnight at 4°C. The plate was washed three times with washing buffer (PBS plus 0.05% Tween-20, PBS-T) and blocked with 2% fish gelatin (Sigma-Aldrich) in PBS for 120 min at RT. Thereafter, the plate was washed three times, and 50 *μ*L of rabbit anti-DMPO serum (1 : 4000, Caymen chemicals, MI) in 1% fish gelatin in PBS was added and allowed to incubate for 60 min at RT. The plate was washed three times with PBS-T, and 50 *μ*L of a goat anti-rabbit IgG (H+L) HRP conjugate (1 : 5000 in washing buffer; Pierce Chemical Co., Rockford, IL) was added and allowed to incubate for 1 h at RT in the dark. The plate was washed with PBS-T and developed by adding 50 *μ*L per well of 3,3′,5,5′-tetramethyl-benzidine (TMB) liquid substrate (Sigma, Inc., St. Louis, MO). The reaction was stopped with 25 *μ*L per well of 1.0 M H_2_SO_4_, and read at 450 nm using an ELx808 microplate reader (BIO-TEK Instruments, Inc., Winooski, VT).

### 2.13. Glutathione (GSH) Competitive Assay

A 50 *μ*L aliquot of tissue homogenate prepared as above described was immediately treated with 50 *μ*L of 10 mM *N*-ethylmaleimide (NEM; Sigma) in PBS and incubated at room temperature for 1 h away from light. The mixture was centrifuged at 11,750 ×g for 10 min at 4°C and the supernatant isolated for analysis. A 96-well ELISA plate was coated with 100 *μ*L per well of 1% BSA (Sigma-Aldrich) in PBS, incubated at 4°C overnight and washed with washing buffer (PBS containing 0.05% Tween-20, PBS-T) using an ELISA plate washer. A 100 *μ*L per well sample of 1.0 mM BS^3^ (0.572 mg/mL; Pierce) in 0.05 M phosphate buffer (pH 8) was added and allowed to incubate for 15 min at RT, followed by the addition of 20 *μ*L per well of 0.025 M GS-NEM (10.8 mg/mL) in PBS (pH 7.4) for 30 min. After incubation for 30 min at RT, the plate was washed and blocked with 100 *μ*L per well of 10 mM glycine (Sigma) in washing buffer for 30 min at RT. The plate was washed, and 50 *μ*L per well of sample were incubated with mouse 8.1-GSH (1 : 50,000 in PBS containing 0.05% Tween-20 and 0.1% BSA; Stressgen). The sample was preincubated for 30 min with 8.1-GSH at RT prior to addition to the plate and once added the sample was allowed to incubate at RT for 1 h. The plate was washed, and 50 *μ*L per well of goat anti-mouse IgG (whole molecule)-peroxidase (1 : 5000 in washing buffer; Sigma) was added. The plate was allowed to incubate in the dark for 1 h at RT; it was then washed and developed with 50 *μ*L per well of 3,3′,5,5′-tetramethyl-benzidine (TMB) liquid substrate (Sigma). The reaction was stopped with 25 *μ*L per well of 1.0 M H_2_SO_4_ and read at 450 nm with an ELx808 microplate reader (BIO-TEK). Standard curve stock glutathione-*N*-ethylmaleimide (GS-NEM) solutions were prepared by slow addition of 3.90 mg (0.0127 mmole) of reduced GSH (Sigma) dissolved in 500 *μ*L of PBS to 1.29 mg (0.0127 mmole) *N*-ethylmaleimide (NEM; Sigma) in 500 *μ*L PBS at RT. The reaction was followed by UV-visible spectroscopy (*λ* 304–315 nm, NEM) until complete. Serial dilutions from the standard stock GS-NEM (5.50 mg/mL, 2.28 × 10^−2^ M) solution were prepared ranging from 2.00 × 10^−4^ M to 6.25 × 10^−6^ M as well as a blank PBS solution without GS-NEM. An aliquot of each solution was then treated with an equal volume amount of 8.1-GSH (1 : 50,000 in PBS containing 0.05% Tween-20 and 0.1% BSA) (solutions were incubated with 8.1-GSH for 30 min at RT prior to addition to the plate), added to the ELISA plate along with the samples, and developed as described earlier. The standard GSH curve was determined by plotting O.D. versus log of the concentration, calculation of the amount of GSH, and determination of GSH concentration, in *μ*g GSH/mg protein.

### 2.14. Measurement of Nitrite Production

NO production was measured as nitrite (NO_2_^−^) concentration by the Griess assay [39]. Griess reagent (50 *μ*L) was added (v/v) to tissue homogenate and standard (NaNO_2_) in 96-well plates. Plates were incubated at room temperature for 10 minutes and read at 550 nm in an ELISA reader.

### 2.15. Protein Assay

The protein concentration of tissue homogenate was tested by using BCA Protein Assay Kit (Pierce). The manufacturer protocol was utilized to perform the assay. In general, the samples were mixed with BCA reagent A (containing mainly bicinchoninic acid) and BCA reagent B (containing cupric sulfate). After 30 min incubation, the sample was read at 570 nm. The sample protein concentration was calculated based on the BSA standard.

### 2.16. Statistical Analysis

Statistical analysis was performed by SigmaStat (Jandeel Scientific, San Rafael, CA). For multiple group comparison, one-way ANOVA or one-way ANOVA on Rank was employed; for two group comparison, *t* test or Mann-Whitney Rank Sum test was used; *P* ≤ 0.05 was considered significant based on selection by the SigmaStat program.

## 3. Results

### 3.1. Danshen as a Daily Food Supplement for 70 Days Did Not Show Any Toxic Effects

Mice on a daily powder diet supplemented with 0.5%, 1%, or 2% Danshen had no significant differences from the control group (0% Danshen) with regard to average daily food consumption and to body weight gain (Table 1). Mice were given the different diets from 3 months of age until the end of all experiments. Danshen as a daily food supplement up to 2% had no obvious toxic effects on BALB/c mice. After *LM* infection, mice lost 2.6–4.9% of their body weight, suggesting there were no major changes in sickness behavior, which affects eating and drinking.

### 3.2. Danshen Did Not Affect the IgG1, IgG2a, or IgE Antibody Response to KLH of BALB/c Mice

After a primary and secondary immunization and having been ingesting Danshen for 56 days, sera were assayed for the levels of IgG1, IgG2a, and IgE anti-KLH. Relative anti-KLH values were obtained by comparing sera with a commercial IgG anti-KLH. Data represent the mean of relative values ± standard deviation (SD). The sera levels of the KLH-specific IgG1, IgG2a, and IgE antibody levels were not significantly altered with any of the supplemental Danshen doses (Table 2).

### 3.3. The Serum Level of IgE Was Inhibited by 0.5% Danshen

**Figure 2:**
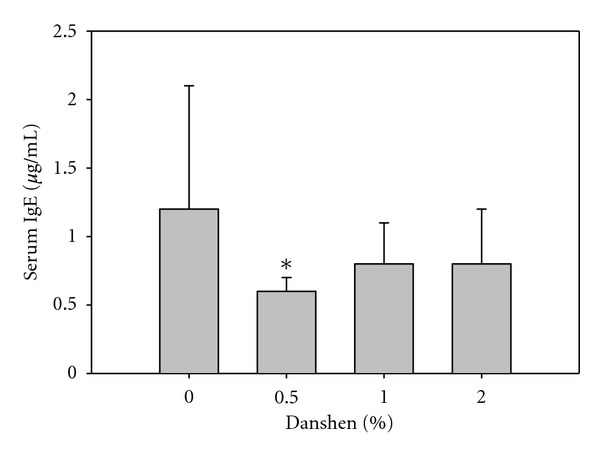
Effects of Danshen on total serum IgE levels. The sera collected from the 0–2% Danshen groups were used for assessment of total serum IgE by ELISA. Results were obtained from five separate experiments for 0% Danshen (*n* = 17); three separate experiments for 0.5% Danshen (*n* = 8); four separate experiments for 1% Danshen (*n* = 12); and three separate experiments for 2% Danshen (*n* = 10). Data are presented as mean ± standard deviation (SD); *designates statistically significant difference (*P* < 0.05) compared with 0% Danshen.

After 56 days of Danshen ingestion, the serum IgE levels were significantly decreased only in the 0.5% Danshen mice (Figure 2).

### 3.4. Danshen Induced a Stronger KLH-Specific DTH Response in 1% Danshen Group

**Figure 3:**
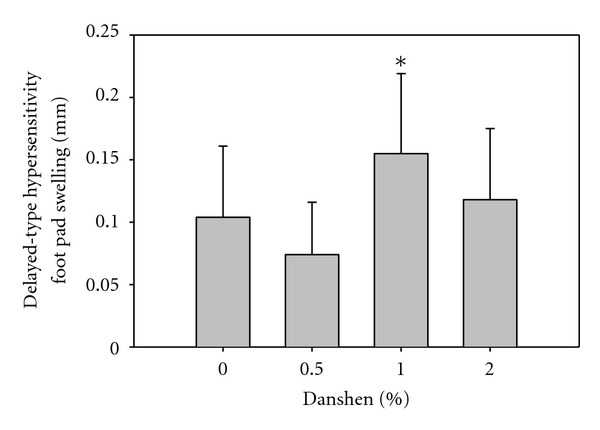
Effects of Danshen on DTH responses. After two immunizations with KLH for assessment of antibodies to KLH, BALB/c mice received a footpad challenge (100 *μ*g KLH in 25 *μ*L saline) 21 days after the second immunization. The DTH response was measured 24 hr later. Results of the 0% (*n* = 16), 0.5% (*n* = 8), 1% (*n* = 11), and 2% (*n* = 9) Danshen are shown as mean ± SD; *indicates that the 1% Danshen group significantly differs from the 0% and 0.5% groups.

After 70 days of Danshen ingestion, the mice that were assayed for antibodies to KLH were challenged in the footpad with 100 *μ*g KLH (25 *μ*L). Mice ingesting the 1% Danshen diet had a significantly enhanced KLH-specific DTH response (0.155 ± 0.064 mm) compared with mice ingesting 0.5% Danshen (0.074 ± 0.042 mm) and 0% Danshen (0.104 ± 0.057 mm) (Figure 3). Data are presented as mean ± SD. Interestingly, ingesting more Danshen (2%) as daily food supplement had no significant effect on KLH-specific DTH response of BALB/c mice.

### 3.5. Danshen Did Not Affect the Serum Cytokine/Chemokine Levels before *LM* Infection

Serum cytokines were analyzed with a commercial Luminex kit. Mouse GM-CSF, IFN-*γ*, IL-10, IL-12, IL-13, IL-17, IL-1*β*, IL-2, IL-4, IL-5, IL-6, JE, TNF-*α*, VEGF were tested. Mouse IL-2, IL-4, VEGF were detectable in all samples. IL-1*β*, IL-5, IL-6 were detected in some samples. However, no significant changes were observed amongst all of the groups.

### 3.6. Danshen Affected the Absolute Numbers of the Leukocyte Subsets in BALB/c Peripheral Blood after *LM* Infection

Peripheral blood was collected from the mice before and after *LM* infection. The absolute numbers of neutrophils, monocytes, lymphocytes (T and B cells), and NK cells were calculated based on four to nine mice from two to four separate experiments. Although before *LM* infection, the numbers of blood neutrophils, monocytes, lymphocytes, and NK cells were not significantly different among all of the groups, at three days after infection, the 0%, 0.5%, and 1% Danshen groups had a significant decrease in number of circulating lymphocytes (Table 3). The 0.5% Danshen group had a significant increased number of NK cells. The 2% Danshen group had no significant decrease of B and T cells, and they had a significant increase in the numbers of monocytes and NK cells. The lack of a loss of lymphocytes and the increases of monocytes and NK cells may have been due to lower numbers of lymphocytes, monocytes, and NK cells prior to infection. Even those these numbers were not significantly different from the other groups.

### 3.7. Danshen Increased Host Defenses against *LM* Infection in Liver and Spleen in BALB/c Mice

**Figure 4:**
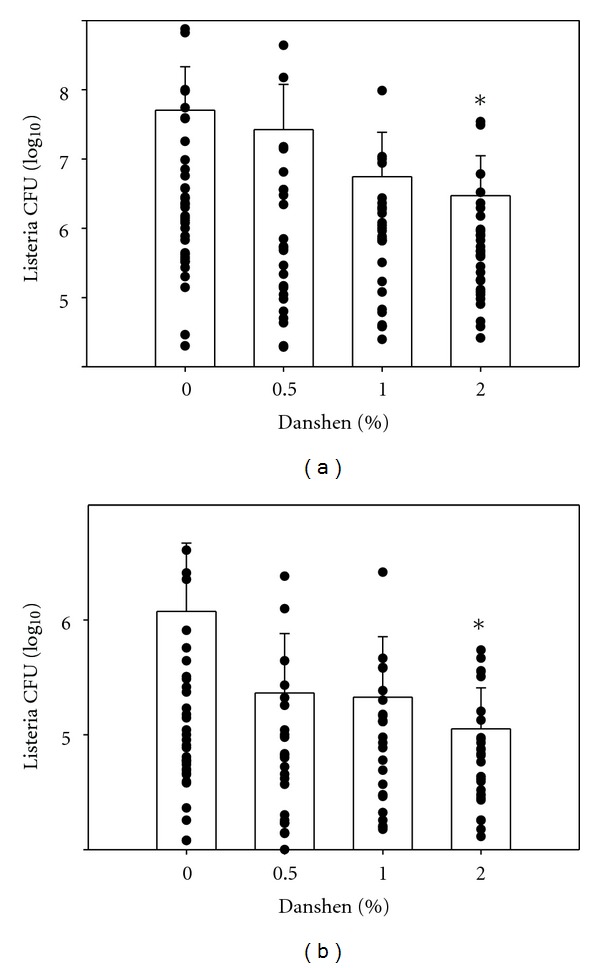
Effects of Danshen on host defenses against *LM* infection in liver (a) and spleen (b). Three days after i.v. injection of 5–8 × 10^3^ CFU, livers and spleens were harvested. The homogenates of liver and spleen were cultured on blood agar plates for quantification of CFU. Results for 0% Danshen (*n* = 36 of 14 separate experiments), 0.5% Danshen group (*n* = 24 of 8 separate experiments), 1% Danshen group (*n* = 26 of 10 separate experiments), and 2% Danshen group (*n* = 30 of 10 separate experiments) are shown as mean ± SD; *indicates statistically significant difference compared to the 0% Danshen group.

After 70 days on the various diets, mice were infected with *LM*. After three days, the mice were sacrificed, and livers and spleens were harvested for enumeration of the viable bacterial burden. Compared to the non-supplemented diet (0% Danshen), only the 2% Danshen diet significantly improved host defenses against *LM*; the 2% Danshen group had decreased bacterial burdens in the liver and spleen (Figures 4(a) and 4(b)).

### 3.8. Danshen Inhibited the Production of Proinflammatory Cytokines after *LM* Infection

**Figure 5:**
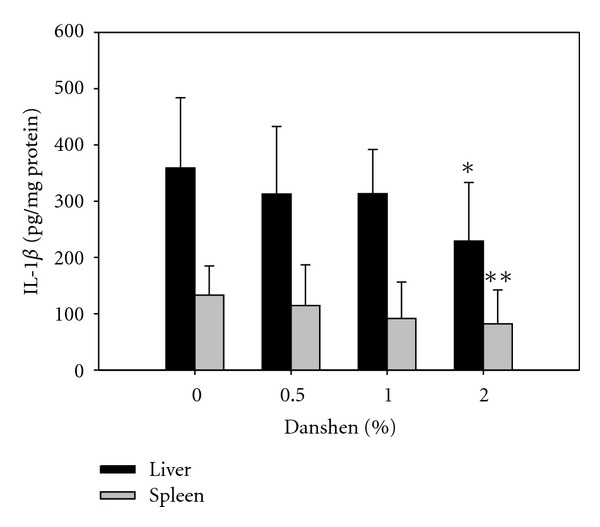
Effects of Danshen on IL-1*β* production in liver and spleen. The homogenates of liver and spleen were tested for IL-1*β* production by ELISA. Results for 0% Danshen (*n* = 14 of 7 separate experiments), 0.5% Danshen (*n* = 11 of 4 separate experiments), 1% Danshen (*n* = 13 of 6 separate experiments), and 2% Danshen (*n* = 15 of 5 separate experiments) are shown as mean ± SD; *(for liver) and **(for spleen) indicate statistically significant difference compared to the 0% Danshen group.

After *LM* infection for three days, the mice were sacrificed. Part of the liver and spleen homogenates were utilized for the measurement of viable *LM* burden. The rest of the tissue homogenates were used to quantify cytokine levels. The 2% Danshen group showed significant inhibition of the production of the early-acting proinflammatory cytokine, IL-1*β* in liver and spleen compared with the 0% Danshen group (Figure 5). The production of the late-acting proinflammatory cytokine HMGB-1 was increased 4 to 10 fold in spleen homogenate over that of liver homogenates (data not shown). However, the production of HMGB-1 was not significantly altered in liver or spleen amongst the Danshen groups. Other cytokines, such as IL-6, TNF-*α*, IFN-*γ*, IL-17, IL-18, IL-33, and TGF-*β* were also tested, but no significant changes were observed (data not shown).

### 3.9. Danshen-Inhibited NO Production in Liver but Not in Spleen

**Figure 6:**
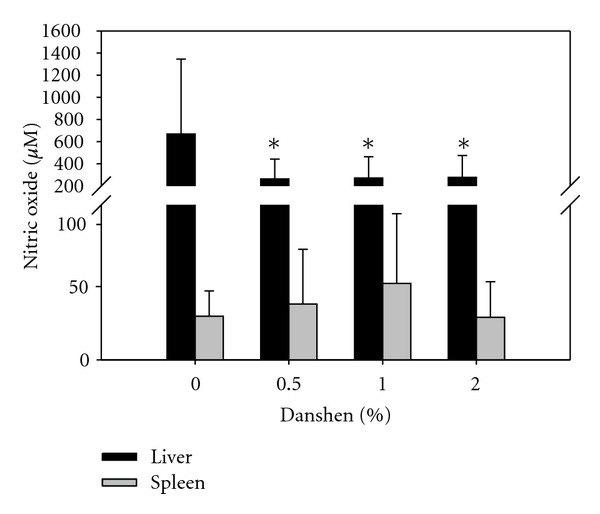
Effects of Danshen on NO production in liver and spleen homogenates. In addition to test cytokine production, the tissue homogenates were measured for NO production. Data for 0% Danshen (*n* = 36 of 14 separate experiments), 0.5% Danshen (*n* = 23 of 8 separate experiments), 1% Danshen (*n* = 26 of 10 separate experiments), and 2% Danshen (*n* = 30 of 9 separate experiments) are shown as mean ± SD; *indicates statistically significant difference compared to the 0% Danshen group.

After *LM* infection, the tissue homogenates also were tested for NO production. NO production in the liver was uniformly inhibited with all doses of Danshen, but no significant changes were observed in spleen (Figure 6).

### 3.10. Danshen Limited Generation of Oxygen Free Radicals in Liver and Spleen after *LM* Infection but Had No Significant Effects on GSH Levels

**Figure 7:**
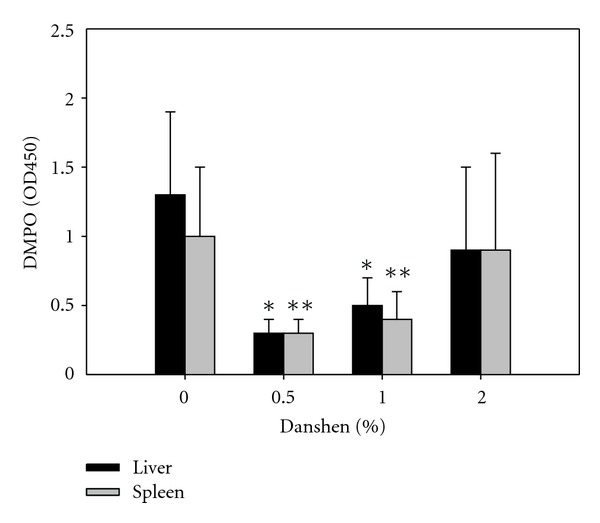
Effects of Danshen on DMPO levels in liver and spleen homogenates. In addition to testing cytokine and NO production, the tissue homogenates were also measured for DMPO levels which indicate oxygen free radicals in tissue homogenates. Data for 0% Danshen (*n* = 11 of 5 separate experiments), 0.5% Danshen (*n* = 6 of 2 experiment), 1% Danshen (*n* = 8 of 3 separate experiments), and 2% Danshen (*n* = 8 of 3 separate experiments) are shown as mean ± SD; *(for liver) **(for spleen) indicate statistically significant difference compared to the 0% Danshen group.

The GSH levels of the tissue homogenates were not significantly altered in any Danshen group after *LM* infection (data not shown). DMPO binds to protein radicals. Therefore, decreasing DMPO indicates less oxygen free radicals existing. On the other hand, the levels of DMPO, which binds to protein radicals and, therefore, is a measure of reactive oxygen species (ROS) modification of proteins, were significantly decreased in the liver homogenates of the 0.5% and 1% Danshen groups and in the spleen homogenates of the 1% Danshen group (Figure 7).

## 4. Discussion

This study was designed to evaluate the immunomodulatory effects of Danshen, which was fed to BALB/c mice as a dietary supplement. At different dietary supplemental doses of Danshen, there were some different immunomodulatory effects. These different effects are suggested to be due to certain compounds being dominant at the different doses of Danshen. The lowest tested dose of Danshen (0.5%) inhibited the serum levels of IgE and inhibited production of NO and generation of protein radicals; it did not affect antibody production, the DTH response, or host defenses against *LM*. Thus, this relatively low dose might be useful for allergies and asthma, especially in that it did not inhibit protective immune responses.

The highest dose used in this study was 2% Danshen; this dose produced no detrimental effects, and it significantly enhanced host defenses against *LM*, so it may be useful for promoting host resistance against pathogens. At 3 days after *LM* infection, the number of peripheral blood monocytes and NK cells were significantly increased with 2% Danshen. The infection induced a significant loss of peripheral blood lymphocytes with the 0%, 0.5%, and 1% Danshen, but not with 2% Danshen. In addition, 2% Danshen inhibited the production of the early-acting proinflammatory cytokine IL-1*β* in the liver and spleen after *LM* infection. Danshen (0.5–2%) inhibited the production of NO in liver but not in spleen after infection, but none of these doses interfered with host defenses against the *LM* infection.

It is evident that IgE plays a critical role in allergy and asthma [40–42]. The 0.5% Danshen dose significantly decreased IgE production in BALB/c mice, suggesting that 0.5% Danshen may have an inhibitory function on an allergic or asthmatic reaction. In fact, our finding is in agreement with Ryu et al., Chio and Kim, and Yang et al. [43–45]. Their research suggested that active components from Danshen had anti-allergic activities; these components were able to directly inhibit mast cell degranulation and interfere with Fc*ε*RI-mediated tyrosine phosphorylation of PLC*γ*2 and MAPK [44]. Usually, the Type 2 (Th2) cytokines IL-4 and IL-13 induce the synthesis of IgE [46–48]. However, IL-13 was not detectable and the amount of IL-4 was not changed amongst all of the groups ingesting Danshen (data not shown), and the absolute numbers of peripheral blood CD4^+^ T cells were not significantly altered (data not shown). Danshen may not inhibit IgE production via any direct effects on helper T cells. Recently, it has been suggested that membrane bond CD23 (Fc*ε*RII) can induce negative signaling for IgE synthesis [40]. Considering the findings of Chio et al. it is possible that Danshen directly affects Fc*ε*RI expressing cells, such as mast cells, with activation of the CD23-induced negative signaling pathway for inhibition of IgE synthesis. Our observation that Danshen decreased IgE production could be a consequence of Danshen's direct effect on mast cells or an activation of the negative signaling pathway for IgE synthesis.

Unlike the influence on the total IgE level, no dose of Danshen modified the antibody response to KLH including the IgE anti-KLH levels, which suggests that the previously described effect on the total IgE level may not have been due to effects on IgE synthesis. It has been suggested that Type 1 (Th1) cytokine, IFN-*γ*, and Th2 cytokines, IL-4 and IL-13 are involved in B-cell class switching. The synthesis of IgG2a is promoted by IFN-*γ* [49, 50], while the synthesis of IgG1 is driven by IL-4 and IL-13 [46–48]. Since B-cell class switching is altered by Th1 or Th2 cytokines and Danshen had no apparent effect on B-cell class switching, it is reasonable to conclude that Danshen had no preference on T-cell Th1 and Th2 skewing. In fact, our serum cytokine/chemokine data are in agreement with this conclusion since the only cytokine that any dose of Danshen affected was that of 2% Danshen on IL-1*β* levels. Additionally, none of the doses of Danshen interfered with antigen-presentation to Th cells, Th cell help for B cells, or B cell activation and production of antibodies since there was no inhibition of antibody production to KLH, a T-cell-dependent antigen.

Host resistance to *LM* infection requires both innate and adaptive immunity. However, based on our previous study, it seems that innate immunity plays a critical role in regulating defenses against *LM* infection [35]. Neutrophils are the first line of defense to control the bacterial growth [51]. The neutrophils are recruited into infectious sites by IL-6 and other factors [52], and then they secret chemokines to attract macrophages to the infectious foci [53]. It has been shown that macrophages are the essential cells in mediating clearance of *LM* [51]. In response to infection, macrophages secret TNF-*α* and IL-12 [54–56], which drives NK cells to produce IFN-*γ*. IFN-*γ* in turn leads to activation of the macrophage and increases their killing capability [51]. Interestingly, DTH is commonly utilized for testing cell-mediated immunity, and only the 1% Danshen dose was able to enhance cell-mediated immunity, but it did not enhance elimination of *LM*. This further suggests that the host defenses against *LM* may be more dependent on innate mechanisms. The 2% Danshen dose, which did enhance killing of *LM*, but did not significantly affect the DTH response may be mainly affecting innate immunity. The enhancement of host defenses with the 2% Danshen was associated with a significant increase in the numbers of increased peripheral monocytes and NK cells after *LM* infection. In addition, 2% Danshen lessened the loss of peripheral lymphocytes after *LM* infection, suggesting that 2% Danshen also has protective effects against the usual *LM*-induced apoptosis of lymphocytes [57]. Although 2% Danshen significantly increased the numbers of peripheral monocytes and NK cells after *LM* infection compared to before *LM* infection, the numbers of each leukocyte subset in peripheral blood were not significantly different among all experiment groups. Additionally, we could not detect any significant increased production of IFN-*γ*, TNF-*α*, or other Th1 types of cytokines in liver or spleen homogenate among all groups at day three after infection. Only IL-1*β* production was significantly inhibited in liver and spleen after *LM* infection. This decreased IL-1*β* production correlated very well with the improved host resistance to *LM* infection in liver and spleen. Our data suggest that decreased production of early proinflammation cytokine IL-1*β* is beneficiary to the host defense. Recently, the published data suggest that mitochondria are involved in innate immune response [58, 59]. Danger signals, environmental stressors, and other factors can trigger mitochondria damage and stress which induce cytokine production, such as cytokine IL-1*β*, IL-18. Here, we found significant inhibition of IL-1*β* production, indicating that 2% Danshen's effect on host defense may be due to successful maintenance of mitochondria homeostasis. In future study, we will examine Danshen's effect on caspase-1 activation by western blot to further explore the mechanism. Unlike previous findings [9, 28, 29], we observed inhibited production of the early proinflammatory cytokine, IL-1*β*, but not the late-proinflammatory cytokine, HMGB-1. This difference could be due to differences with the* in vivo* models, in that the previous reports examined the effects of Danshen on survival rates after LPS-induced sepsis, while we investigated bacteria clearance *in vivo*.

NO is an important biomolecule with multiple functions. It has been reported that NO is required for *LM* clearance in SCID mice [60]. However, NO can disturb endopasmic reticulum (ER) functions causing ER stress and induce cell apoptosis, and a large amount of NO is toxic to the host [61]. Liver levels of NO were uniformly inhibited with all doses of Danshen. The lower NO production may be associated with Danshen's antioxidant capacity. NO can be an indicator for oxidative stress-related tissue damage. The effect of Danshen on NO synthesis was organ specific, since we only observed the inhibition of NO in liver and not in spleen. The liver is a major site for *LM* clearance as well as NO production; however, an excess of inflammatory cytokines and NO, which often go hand in hand with early innate immune responses, may antagonize some protective mechanisms against *LM*. Thus, Danshen's antioxidant activities may aid immunity against the *LM* infection by lowering the stress level. In order to fight bacteria, innate immune cells form reactive oxidants [62], at the same time, the innate enhanced inflammation elevates oxidative stress to host cells [62, 63]. It has been reported that active compounds of Danshen are able to activate antioxidant defense enzymes [8, 9, 17, 21, 22], scavenge oxygen free radicals [18–20, 30, 31], reduce ROS formation [6, 7, 9, 21], and prevent intracellular GSH depletion [7]. Although we could not detect any significant GSH changes in liver or spleen between the groups, oxygen free radicals were significantly decreased with 0.5% and 1% Danshen in both liver and spleen.

Previously, we had reported that a physical/psychological stress immediately before *LM* infection interferes with the development of immunity against *LM* and the stress lead to an elevation of proinflammatory cytokines and NO [64, 65]. Moreover, this stress-induced enhancement of NO and inflammatory cytokines also causes loss of cellular thiols, which correlated with inhibition of host defenses [66]. Since the physiological benefit of Danshen has long been considered its reduction of stress, the significantly enhanced host defense is likely because 2% Danshen is able to successfully control the *LM*-infection-induced stress. This improved host defense which does not appear to be due to the mice ingesting 2% Danshen having more circulating leukocytes compared to 0% Danshen, because we did not detect any significant increase of those cells before or after *LM* infection compared to 0% Danshen group. However, at this point, we still cannot rule out the possibility that more cells could be trafficking to the organs. In addition, unlike classic bacteria fighting, this enhanced host resistance was not correlated with increased production of Th1 type of cytokines. Taken together, 2% Danshen promoted host defense is very unique. It is possible because of the balance between 2% Danshen's immunostimulatory ability and its anti-inflammation and antioxidative activity, which control *LM* growing at the beginning of the infection and protect immune cells from excessive inflammation and oxidative stress under infection conditions, thus, promoting better host defense.

Another possible explanation for how Danshen improved host defense is that Danshen stabilizes intracellular Ca^2+^ storage in host cells to help immune cells against *LM* infection. Ca^2+^ signaling plays an important role for effectors cells of the immune system. It has been reported that the cytosolic Ca^2+^ level is elevated with oxidative stress [67–69], and an elevated intracellular Ca^2+^ level is associated with immune cell apoptosis [70, 71]. Listeriolysin O (LLO), the pore-forming toxin of *LM,* not only triggers Ca^2+^ signaling involved in many host responses [72, 73], but also depletes host intracellular Ca^2+^ storage to the benefit of *LM* survival in host cells [74]. Danshen is capable of stabilizing intracellular calcium [15, 16]. Therefore, it favors host cells.

Taken together, the different doses of Danshen show different immunomodulatory effects in this study. Our data may explain why various activities of Danshen had been described in previous studies. This study may also provide a useful guide for future Danshen studies. For example, low dose (0.5%) of Danshen inhibited total IgE production; therefore, low dose of Danshen can be utilized for antiallergy or antiasthma study. Using a medium (1%) dose of Danshen can be a strategy to investigate cell-mediated immunity and antioxidative activity, while the higher (2%) dose of Danshen seems suitable for studying host defense and anti-inflammatory activity.

## Figures and Tables

**Table 1:** Effects of Danshen on average body weight change and daily food uptaken in BALB/c mice.

Supplemented daily diet	Body weight at d0 (grams)	Body weight at d70 (grams)	Body weight increase %	Daily food/per mouse (grams)
0% Danshen	27.1 ± 0.67	29.5 ± 0.44	9.6 ± 1.47	4.5 ± 0.09
0.5% Danshen	27.2 ± 0.59	30.0 ± 0.61	10.4 ± 1.44	4.3 ± 0.17
1% Danshen	29.2 ± 0.41	29.8 ± 0.28	9.6 ± 1.45	4.4 ± 0.03
2% Danshen	27.0 ± 1.01	30.0 ± 0.29	11.0 ± 0.78	4.3 ± 0.03

Mice were weighed before ingesting Danshen at day 0 (d0) and at d70. Daily food/per mouse was calculated by total amount of food uptaken divided by total days and total number of mice. The data of normal food group were calculated from 20 mice of 6 experiments; the data of 0.5% Danshen group were calculated from 14 mice of 4 experiments; the data of 1% Danshen group were calculated from 16 mice of 5 experiments; and the data of 2% Danshen group were calculated from 15 mice of 4 experiments. The data are presented as mean ± SEM. There are no significant differences detected among all experiment groups.

**Table 2:** Effects of Danshen on an amnestic antibody response.

Supplemented daily diet	IgG1 anti-KLH	IgG2a anti-KLH	IgE anti-KLH
0% Danshen	6595 ± 1050	920 ± 148	1.6 ± 0.18
0.5% Danshen	7327 ± 2194	953 ± 242	1.1 ± 0.23
1% Danshen	6884 ± 1222	929 ± 157	1.3 ± 0.12
2% Danshen	7407 ± 1684	1113 ± 260	1.4 ± 0.20

The data are presented as mean ± SEM of ELISA values calculated as described in Methods.

There are no significant differences detected among all of the groups.

The numbers of mice per group were as follows: 0%, *n* = 17; 0.5%, *n* = 8; 1%, *n* = 12; and 2%, *n* = 10.

**Table 3:** Danshen effects on the numbers of peripheral blood leukocyte subpopulations before and after *LM* infection.

Danshen (% of daily diet)	Neutrophils (cells/*μ*L)			Monocytes (cells/*μ*L)			Lymphocytes (cells/*μ*L)			NK cells (cells/*μ*L)		
	Preinfection	Postinfection	*P* value	Preinfection	Postinfection	*P* value	Preinfection	Postinfection	*P* value	Preinfection	Postinfection	*P* value
0	2747 ± 279	2038 ± 908	ns	717 ± 146	885 ± 371	ns	4865 ± 694	1258 ± 523	0.018	193 ± 32	214 ± 57	ns
0.5	1903 ± 147	2928 ± 1462	ns	798 ± 95	1213 ± 425	ns	4509 ± 594	1841 ± 508	0.014	136 ± 26	653 ± 118	0.029
1	3022 ± 890	2128 ± 399	ns	912 ± 134	1051 ± 134	ns	4848 ± 737	1242 ± 246	0.004	219 ± 82	543 ± 191	ns
2	2749 ± 1344	2414 ± 247	ns	484 ± 71	1220 ± 253	0.012	3419 ± 518	1890 ± 460	ns	104 ± 22	321 ± 70	0.006

After ingesting 0–2% Danshen for 70 days, peripheral blood was collected before and after *LM* infection. The sample was prepared in a TruCOUNT tube and analyzed by flow cytometry. The absolute number of neutrophils, monocytes, lymphocytes, and NK cells was calculated based on four to nine mice from 2–4 separated experiments. Data are presented as mean ± SEM.
